# Work Psychology and Occupational Health: An Editorial

**DOI:** 10.3390/ijerph22010100

**Published:** 2025-01-13

**Authors:** Masahito Fushimi

**Affiliations:** Akita University Health Center, Akita University, 1-1 Tegatagakuen-machi, Akita 010-8502, Japan; fushimi@gipc.akita-u.ac.jp or mfushimi8@gmail.com

Globally, the COVID-19 pandemic has severely impacted workers’ health, particularly their mental well-being [1,2,3,4]. While numerous studies have highlighted significant consequences for workers’ well-being, there remains a lack of thorough evaluation and understanding of the pandemic’s effects on employees and businesses, particularly concerning the psychological impacts and coping methods [5,6,7,8,9,10]. We are thrilled that this Special Issue includes contributions examining the impact of the pandemic on the work environment, the connection between the pandemic and workers’ psychological effects, and strategies for maintaining workers’ health—particularly mental health—during this time. These studies underline the necessity of recognizing the current circumstances and implementing workplace and individual strategies to alleviate stress and enhance resilience during the pandemic [Contributions 1–10].

Several papers in this Special Issue focused on essential topics such as healthcare workers’ stress and psychological impact during the COVID-19 pandemic [Contributions 1, 2, 4]. Considering these factors, Bellehsen and his team explored how to modify the stress first aid model for healthcare workers on the frontlines during COVID-19 [Contribution 1]. They observed that introducing stress first aid could equip workplaces and individuals with essential skills to alleviate stress and foster resilience. These skills are beneficial during periods of healthcare-related stress. Discussing strategies for stress reduction and enhancing resilience is crucial for effectively designing and delivering healthcare services. Safiye et al. presented their cross-sectional study on mentalizing, resilience, and healthcare workers’ mental health amid the COVID-19 pandemic [Contribution 2]. Their research importantly seeks to highlight key insights regarding the factors influencing depression, anxiety, and stress, emphasizing the critical need for developing and applying strategies that promote resilience and improve the mentalizing abilities of healthcare workers. Lee and colleagues present their exploratory study on nurses’ emotions related to COVID-19 following their experiences with SARS [Contribution 4]. From discussions with nurses who experienced both SARS and COVID-19, it became clear that the media is a vital resource during disease outbreaks. Government departments need to exercise their expertise to utilize media channels effectively. For front-line nurses, it is crucial to grasp the public’s reaction to the disease, receive on-the-job training and guidelines, and have adequate support administration.

The COVID-19 pandemic has had a psychological impact on workers other than healthcare workers, including in education and research [11,12,13,14]. Bearing this in mind, Padmanabhanunni and his team demonstrate in their study the factors leading to and the psychological impacts of teacher burnout during the COVID-19 pandemic [Contribution 3]. The authors highlighted that the dimensions of burnout are strong predictors of psychological well-being indicators, such as depression, hopelessness, anxiety, and life satisfaction. They stress that intervention strategies to lower burnout should equip teachers with sufficient job resources to help them cope with the demands and stressors of their roles. Ramos-García and colleagues presented their exploratory analysis of university employees, examining the ergonomic factors affecting job satisfaction and occupational health during the SARS-CoV-2 pandemic through a structural equation model [Contribution 7]. They noted that organizational and physical factors positively impact job satisfaction. They determine that the findings enhance understanding of ergonomic factors and bolster educational institutions’ sustainability efforts.

Moreover, the SARS-CoV-2 pandemic has highlighted the importance of considering the work environment as an ongoing influence, even after the crisis. This awareness has sparked increased interest in exploring new working arrangements, particularly remote work [Contribution 10], and research into the psychological impact on workers greatly affected by the pandemic, especially those involved in emotional labor [Contributions 5, 6]. Given this context, Cheung’s study demonstrates the practical aspects of managing workplace well-being in post-pandemic remote work settings [Contribution 10]. He emphasizes that staff well-being is vital to managing organizational change. Senior management must implement sensible measures to improve occupational safety in their work-from-home (WFH) policies, following practical recommendations.

Additionally, he suggests that by adopting the proposed evidence-based practices for WFH initiatives, senior management can significantly enhance the overall workplace well-being of employees in the post-pandemic era. Cheng and colleagues delved into emotional labor with two contributions [Contributions 5, 6]. First, they examined how psychological empowerment mediates the relationship between transformational leadership and emotional labor [Contribution 5]. The authors emphasize that these findings enhance our comprehension of transformational leadership’s impact on front-line employees’ emotional labor. Additionally, they highlighted that the perceived external prestige of these employees affects their emotional labor via organizational identification and impression management motives, with the influence of each pathway varying based on the perceived organizational support [Contribution 6].

Several of our studies specifically examined the recommendations for mental health professionals and education policymakers to tackle the issues related to educational leadership. In particular, the case study by Wang and colleagues investigated the challenges faced by academic leaders, highlighting the essential factors required to ensure a safe workplace [Contribution 8], and the case study by Zhang and colleagues explored leadership [Contribution 9]. These topics are crucial for workplace mental health even after the pandemic. Wang and colleagues presented a case study examining occupational stress among doctoral supervisors in Chinese higher education institutions [Contribution 8]. They offered their recommendations on how mental health professionals and educational policymakers can tackle the concerns of doctoral supervisors by analyzing stress triggers. Zhang and team detailed the impact of safety management leadership on employees’ safety performance based on a case study conducted in China’s mining sector [Contribution 9]. They pinpointed key dimensions by exploring the correlations between leadership safety practices and safety outcomes.

In conclusion, the contributions to this Special Issue offer valuable insights for conducting research and evaluation within this context, informing future inquiries on the topic. This compilation features diverse studies from an international pool of authors, enhancing the perspectives presented in this body of work. I am especially pleased that early career researchers heeded our invitation to submit their work, highlighting our commitment to facilitating their publishing efforts. We appreciate the mentorship provided to them by seasoned colleagues and the support from the editorial team at IJERPH. I send a heartfelt thank you to every author who responded to our call for papers and our diligent independent peer reviewers who provided critical feedback. I also acknowledge the thousands of readers of this Special Issue. Lastly, I extend my gratitude to all those involved in the research highlighted here, whose contributions deepen our understanding of this vital field.

I look forward to potentially launching a second edition influenced by this collection, but for now, I hope you find joy in exploring this Special Issue.
